# Beat the Clock: Assessment of Night Eating Syndrome and Circadian Rhythm in a Sample of Greek Adults

**DOI:** 10.3390/nu16020187

**Published:** 2024-01-05

**Authors:** Anastasia Blouchou, Vasiliki Chamou, Christos Eleftheriades, Dimitrios Poulimeneas, Katerina-Maria Kontouli, Konstantinos Gkiouras, Alexandra Bargiota, Kalliopi K. Gkouskou, Eirini Rigopoulou, Dimitrios P. Bogdanos, Dimitrios G. Goulis, Maria G. Grammatikopoulou

**Affiliations:** 1 Department of Nutritional Sciences and Dietetics, Faculty of Health Sciences, International Hellenic University, Alexander Campus, Sindos, GR-57400 Thessaloniki, Greece; anastasiablouchou@gmail.com (A.B.); xamouvasiliki@gmail.com (V.C.); christos344@gmail.com (C.E.); 2 Department of Nutritional Science and Dietetics, School of Health Sciences, University of the Peloponnese, GR-24100 Kalamata, Greece; 3 Department of Primary Education, School of Education, University of Ioannina, GR-45110 Ioannina, Greece; 4 Unit of Immunonutrition and Clinical Nutrition, Department of Rheumatology and Clinical Immunology, Faculty of Medicine, School of Health Sciences, University of Thessaly, Biopolis, GR-41223 Larissa, Greece; 5 Department of Endocrinology and Metabolic Diseases, Faculty of Medicine, University of Thessaly, Biopolis, GR-41223 Larissa, Greece; 6 Laboratory of Biology, School of Medicine, National and Kapodistrian University of Athens, GR-11527 Athens, Greece; gkouskoukal@med.uoa.gr; 7 Genosophy, 1 Melissinon and Damvergidon Street, GR-71305 Heraklion, Crete, Greece; 8 Department of Medicine and Research Laboratory of Internal Medicine, National Expertise Center of Greece in Autoimmune Liver Diseases, Larissa University Hospital, Biopolis, GR-41334 Larissa, Greece; 9 European Reference Network on Hepatological Diseases (ERN RARE-LIVER), Larissa University Hospital, Biopolis, GR-41334 Larissa, Greece; 10 Unit of Reproductive Endocrinology, 1st Department of Obstetrics and Gynecology, Aristotle University of Thessaloniki, GR-54124 Thessaloniki, Greece

**Keywords:** chrononutrition, chronotherapeutic interventions, chronobiology, circadian misalignment, sleep disruption, obesity, metabolic disturbance, insufficient sleep, eating disorder

## Abstract

The night eating syndrome (NES) is characterized by excessive food intake during the evening and night hours, with 25% of the daily intake being consumed post-dinner, paired with ep-isodes of nocturnal food intake, at a frequency of more than twice weekly. The NES has been associated with a misaligned circadian rhythm related to a delay in overall food intake, increased energy and fat consumption. The present cross-sectional study aimed to assess NES in a Greek population and evaluate possible links between NES and chronotype. NES was assessed using the Night Eating Questionnaire (NEQ), and circadian rhythm, sleep and mood were evaluated with the Sleep, Circadian Rhythms, and Mood (SCRAM) questionnaire. A total of 533 adults participated in the study. A relatively high prevalence of NES was revealed, with more than 8.1% (NEQ ≥ 30) of the participants reporting experiencing NES symptoms, depending on the NEQ threshold used. Most participants had the intermediate chronotype. NEQ score was positively associated with the morning chronotype, and SCRAM was negatively related to “Good Sleep”. Each point increment in the depression score was associated with 6% higher odds of NES. The early identification of NES gains importance in clinical practice, in a collective effort aiming to reduce NES symptomatology and its detrimental health effects.

## 1. Introduction

The night eating syndrome (NES) was first identified approximately 70 years ago by Stunkard et al. [1] as a distinct food intake pattern mainly observed among patients with obesity. According to the latest version of the Diagnostic and Statistical Manual for Mental Disorders (DSM-V) [2], NES is classified as an Other-Specified Feeding or Eating Disorder (OSFED), characterized by an excess in food intake during the evening and night hours, at a level where approximately 25% of the daily intake is consumed at evening time (post-dinner), paired with episodes of nocturnal food intake, occurring at least twice a week [3]. Body weight accumulation, overweight and obesity, poor sleep quality and depressed mood are common in individuals with NES, paired with an urge to eat upon nocturnal awakenings [4,5,6,7,8]. Age, sex or income do not appear to be associated with the development of NES [8].

On the other hand, NES has also been associated with a heightened eating disorder (ED) pathology, emotional eating [8], a misaligned circadian rhythm related to a chronological delay in overall food consumption during the day, as well as delayed circadian rhythms for the total caloric, fat and carbohydrate intake [5]. Recent data from Italy have also associated NES with the eveningness chronotype [9].

A link has been established between the circadian system and energy metabolism at the central and peripheral clock levels [10]. The circadian (*circa* “about” and *diem* “day”) system consists of a multi-oscillator network synchronizing most behavioral and physiological functions with the environment, including hunger, time of food intake, metabolism and eating behavior [11,12,13,14]. Various oscillators (timers) have been shown to regulate the circadian system, with the central one being the suprachiasmatic nucleus (SCN), responsible for the sleep–wake cycle, core body temperature, as well as the release of several hormones [15,16]. The SCN is the “master clock”, the central operation unit synchronizing the circadian clock to that of the outside world [16].

The classification of the circadian rhythm encompasses three distinct chronotypes, namely the morning, intermediate and evening types [17]. Morning-type individuals are considered as “phase-advanced”, tending to sleep and waking up early, accomplishing the peak of their mental and physical performance in the mornings, whereas evening types achieve their peak towards the end of the day, preferring later bed and wake-up schedules [16,17]. Individual circadian expression varies and is mainly the confluence of hereditary and environmental influences, as well as sex and age [14,18]. Furthermore, the expression of gene-coding enzymes pivotal in metabolism is, in part, regulated by a circadian mechanism [10,19,20]. Once sleep is disrupted, several factors contribute to an increased food intake, including changes in the levels of appetite regulation hormones like leptin, ghrelin and peptide-YY, which, in turn, increase hunger and appetite [21,22,23] and, by inference, influence the choice and intake of food and the risk of obesity [23]. In parallel, in cases of circadian misalignment and insufficient sleep, specific brain regions stimulated by food are activated [10,24,25] and the endocannabinoid system is also altered [26]. As a result, body mass index (BMI), metabolic disorders and sleep disturbance are strongly correlated [27,28].

When a mismatch occurs between the environment’s endogenous rhythm and time cues, chronodisruption is manifested, inducing changes in the circadian system oscillators in peripheral tissues [16,29]. Chronodisruption may involve loss of synchronization, lower rhythm amplitudes or phase misalignment between the SCN and the peripheral circadian clocks [29]. This circadian desynchrony may be causal and detrimental to the development of metabolic manifestations, including greater waist circumference denoting central obesity, increased blood pressure levels and elevated fasting blood sugar and insulin concentrations [30]. In parallel, a hereditary component has also been identified affecting sleep duration and the intake of energy and macronutrients, as shown in individuals harboring single nucleotide polymorphisms (SNPs) mainly of the circadian locomotor output cycles kaput (CLOCK) gene [31,32,33,34]. The CLOCK and a few additional genes, including period (PER), rev-erb/nuclear receptor subfamily 1 group D (NR1D), brain and muscle ARNT-like (BMAL) and cryptochrome (CRY), have been suggested to form the “molecular circadian clock” [35].

As research on the NES is gaining ground, the present cross-sectional study aimed to assess NES symptomatology in a Greek population and evaluate possible links between NES and chronotype. Understanding the factors associated with NES and the possible links between NES and chronotype might help us manage the symptoms more easily, and design specific chronotherapeutic interventions aiming to reduce NES symptomatology and its detrimental health effects.

## 2. Materials and Methods

### 2.1. Participant Recruitment and Characteristics

Participants were recruited online through social media groups using calls for participation in the survey. Data collection took place from May–July 2021. The initial sample consisted of 536 adults, but 3 participants were excluded from the study due to missing data. Thus, the final sample involved 533 adults residing in Greece and Cyprus. Characteristics of the sample are presented in Table 1.

### 2.2. Ethical Permission

Permission for the study was granted by the Ethics Committee of Aristotle University’s Medical School (4.589/21-02-2022). Additionally, each participant provided consent prior to participation by ticking a box situated at the beginning of the questionnaire. All data were collected anonymously, without any identifying information being recorded.

### 2.3. Assessment of NES

NES was assessed in the sample using the Night Eating Questionnaire (NEQ) [36], a short, validated tool for evaluating the psychometric properties and severity of NES. The NEQ consists of 14 questions, each with five possible answers, most (except from one), on a 0–4 Likert scale, assessing four core factors, namely (i) nocturnal ingestion, (ii) evening hyperphagia, (iii) morning anorexia and (iv) mood/sleep quality. Each question provides a score ranging between 0 and 4, adding to the total NEQ score, which spans between 0 and 52 [36].

Greater NEQ scores indicate higher NES tendencies and a score exceeding 25 points is considered to be confirmatory of NES [36]. Aside from the ≥25 threshold, a score of ≥30 has been suggested for identifying individuals with NES. The former threshold (≥25) has high sensitivity, whereas the latter (≥30) has high specificity [36].

### 2.4. Circadian Rhythm, Sleep and Mood

The participants’ circadian rhythm, sleep and mood were evaluated with the Sleep, Circadian Rhythms, and Mood (SCRAM) questionnaire [37]. The tool consists of three 5-item scales assessing depressed mood, morningness and good sleep during the past two weeks. Each scale contains one reverse-scored item. All items are measured on a 6-point Likert-type scale, ranging from 1 (Strongly Disagree) to 6 (Strongly Agree). A scale score is derived by summing ratings after reverse scoring, wherever required [37]. The range for each scale is between 5 and 30.

Research has evaluated the construct validity and reliability of the SCRAM as a measure of diurnal preference, sleep quality and depressed mood. Furthermore, SCRAM scores have been shown to correlate well with objective sleep–wake behavior measures, using actigraphy, having a strong test–retest reliability (r = 0.73 to 0.83) [38].

### 2.5. Translation of the Questionnaires in the Greek Language

Approval for translation and use of the NEQ was granted by its main author, Professor Kelly C. Allison, after personal communication. Similarly, approval for the translation, adaptation and use of the SCRAM questionnaire was provided by Dr. Jamie E.M. Byrne through email communication.

For the translation and cultural adaptation of the NEQ and the SCRAM questionnaires into Greek, the four-step forward–backward process was applied [39], with permission from the authors of the original tools [36,37]. A total of six bilingual experts (D.G.G., M.G.G., D.P.B., E.R., K.G. and D.P.) participated in the forward–backward translation process.

### 2.6. Anthropometry

Body weight and stature of participants were self-reported, and BMI was calculated for all (body weight [kg]/height [m^2^]). Weight status was assessed using the World Health Organization (WHO) thresholds for BMI, classifying underweight (BMI < 18.5 kg/m^2^), normoweight (18.5 kg/m^2^ ≤ BMI < 25 kg/m^2^), overweight (25 kg/m^2^ ≤ BMI < 30 kg/m^2^) and simple obesity (BMI ≥ 30 kg/m^2^) in adults [40].

### 2.7. Statistical Analyses

Statistical analyses were performed using Jamovi (Version 2.3.21.0) [41] and R language (Version 4.2.2) [42]. The normality of data was graphically explored with Q-Q plots. As all continuous variables were normally distributed, they are presented as means ± standard deviations (SD). Categorical variables are displayed as counts with their relative frequencies. One missing variable in a questionnaire was imputed.

Sex-related differences between continuous variables were explored with independent samples *t*-tests. Differences between categorical values were explored using the chi-square test. Associations between the NEQ and the SCRAM questionnaire (total and sub-scales) were explored with logistic regression (NES as the dependent variable); three models were employed: a crude model, a model adjusted for sex, age and tertiary education, and a model further adjusted for BMI. The level of significance was set at 5%.

The internal reliability of the instruments was assessed with the Cronbach α for ordered variables. Cronbach’s α indicates how closely related a set of items is as a group; it ranges between 0 and 1, with values >0.6 suggesting an acceptable level of reliability and values >0.8 indicating a very good fit [43].

## 3. Results

### 3.1. Translation and Validation of the Questionnaires

The calculated internal reliability of the NEQ was acceptable (Cronbach’s α = 0.65), similar to previous research [36]. The Cronbach α values for the dimensions of the NEQ for evening hyperphagia (α = 0.52) and mood/sleep quality (α = 0.49), however, indicate moderate reliability. The Cronbach’s α was lower than the acceptable threshold for the dimension of morning anorexia and nocturnal ingestions.

On the other hand, the internal validity of the SCRAM questionnaire was relatively low (Cronbach α = 0.44). The three dimensions of the SCRAM questionnaire, however, achieved better reliability. The “Good sleep” domain had a Cronbach α = 0.6, “morningness” showed an α = 0.56 and “depressed mood” exhibited an α = 0.76, indicating a very good fit.

**Figure 1:**
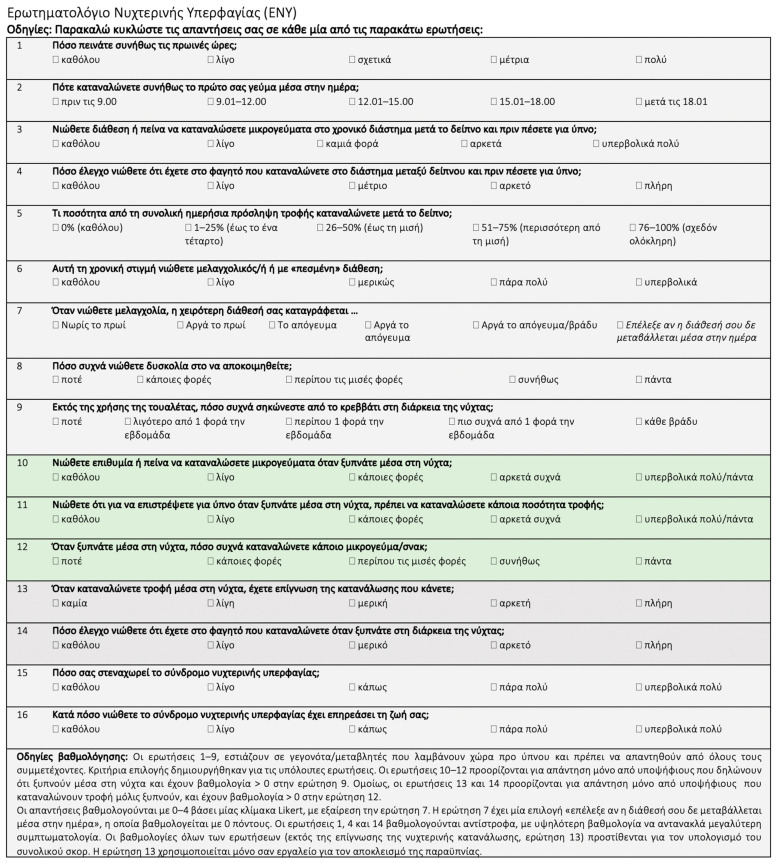
Translation and adaptation of the NEQ [36] for the identification of NES in the Greek language. NEQ, Night Eating Questionnaire [36]; NES, Night Eating Syndrome.

**Figure 2:**
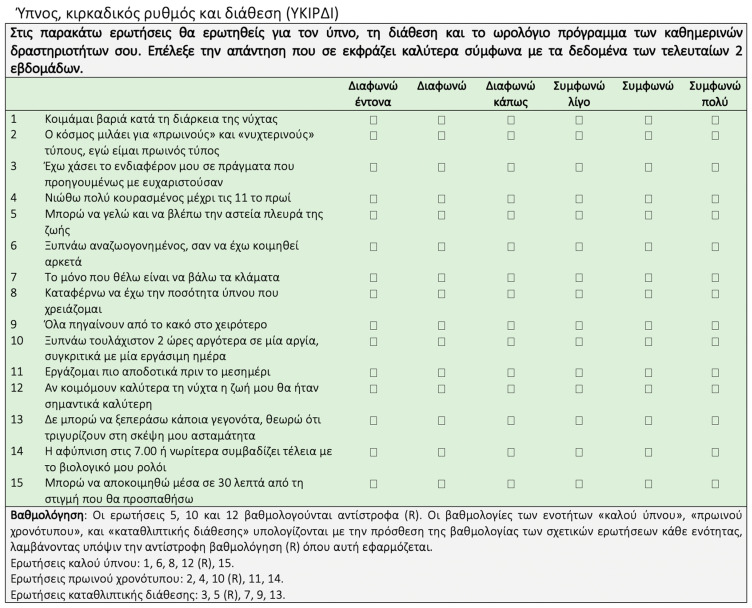
Translation and adaptation of the SCRAM questionnaire [37] in the Greek language. SCRAM, Sleep, Circadian Rhythms, and Mood.

The translation and adaptation of the NEQ and the SCRAM questionnaires into Greek are presented in Figure 1 and Figure 2, respectively.

### 3.2. NEQ and SCRAM Results of the Sample

Table 2 details the results of the NEQ and SCRAM questionnaires. In the total sample, the mean NEQ was 18.0 ± 7.4 (ranging between 3 and 50), with no differences being observed between sexes. NES was diagnosed in 17.8% (NEQ threshold ≥ 25) and 8.1% (NEQ threshold ≥ 30) of the sample.

The mean total SCRAM score of the sample was moderate (49.7 ± 7.9, range 29 to 74), indicating the intermediate chronotype for most participants. Regarding SCRAM subscales, the only observed difference between sexes involved the women who reported a greater frequency of depressive symptoms compared to the men (*p* ≥ 0.001).

### 3.3. NEQ and SCRAM Results by Weight Strata

As detailed in Table 3, no differences were noted between the total NEQ and SCRAM raw scores in different BMI categories (*p* > 0.05 for all). Accordingly, no differences were observed in the prevalence of NES by BMI strata, although a non-significant trend towards greater NES-positive screening was noted among participants with overweight and obesity, compared to those with normal body weight. The sub-analysis performed according to the sex of participants produced similar results.

To further explore the relationship between BMI and NES, we employed a linear regression model (NES, the dependent variable; BMI, the independent variable). No association was observed between NES and BMI (B = 0.111, SE = 0.068, *p* = 0.105). Similarly, no associations were observed in the sub-analysis according to the sex of participants (men, B = 0.113, SE = 0.144, *p* = 0.435; women, B = 0.100, SE = 0.081, *p* = 0.214).

### 3.4. Relationship between NEQ and SCRAM

The association between NES and SCRAM, along with its subscales, is presented in Table 4. When NES was defined as an NEQ score ≥ 25, a marginal trend for an inverse relationship between the total SCRAM and “Good Sleep” was observed. A similar association between NES and the “depression” subscale was noted; each 1-point increment in the depression score was associated with 6% higher odds of having NES. When NES was defined as an NEQ score ≥ 30, a positive association with “morningness” was observed. No other significant associations were detected.

Additionally, we performed the same analyses in the sub-group of participants with simple obesity (BMI ≥ 30 kg/m^2^). With regards to having an NEQ ≥ 25, the results previously observed in the total sample of the study were not replicated [NEQ ≥ 25 vs. Good Sleep: OR = 1.02 (95%CI 0.90–1.15); NEQ ≥ 25 vs. depression: OR = 1.03 (95%CI 0.92–1.15)]. When the more stringent threshold for NES was applied (NEQ ≥ 30), the positive relationship between NES and morningness remained significant, yet only in the model adjusted for age, sex and having attained tertiary education [NEQ ≥ 30 vs. morningness: OR = 1.25 (1.01–1.54)].

## 4. Discussion

The present study revealed a relatively high prevalence of NES. NEQ score was positively associated with the morning chronotype, and SCRAM was negatively related to “Good Sleep”. Each point increment in the depression score was associated with 6% higher odds of NES.

More than 8.1% of the participants herein exhibited NES symptoms depending on the threshold used to identify NES symptomatology. In the United States, the prevalence of NES in the general population reaches 1.5% [44], whereas in Germany, NES affects 1.1% [45] of the inhabitants, compared to 1.5% of the Japanese [46], 0.9% of the Australian population [47], 10% of the adult Turks [6] and 14.4% of people living in Pakistan [48]. One study using the NEQ reported a 54% prevalence among adults in Turkey; however, researchers applied an arbitrary threshold of NEQ ≥ 20 for the classification of NES [49]. When the NEQ score ≥ 25 threshold was applied, 17.8% of the participants exhibited NES. According to Kaur et al. [8], when the cut-off is decreased from 30 to 25 points, the prevalence of NES within the examined populations is more than doubled. The “25” cut-off for the NEQ has a positive predictive value (PPV) of 40.7%, indicating a 40.7% chance that a patient who screened positive actually has NES [36,50]. On the other hand, a NEQ score ≥ 30 is a stronger predictor of NES, with a PPV of 72.7% [36,50]. Thus, using the NEQ score does not diagnose NES, as NEQ has a PPV of 44.7–72.7%, depending on the applied threshold [36,50]. This means that, depending on the threshold applied, participants who screen positive have a 44.7–72.7% chance of having NES. Thus, screening positive does not always coincide with NES.

In the present study, a trend towards greater NES-positive screening was noted among participants with overweight and obesity compared to those with normal body weight. Research has revealed that more than half (55%) of individuals with obesity seeking bariatric surgery report NES symptoms [51]. Although a greater NES prevalence exists in patients with obesity (10.1–27%) [44,52,53,54], not all individuals with NES symptomatology are, in fact, obese [48,55,56,57,58,59,60,61]. Furthermore, research using the NEQ has revealed a greater NES prevalence among patients with mental illness (25%) [62].

The relatively high proportion of participants in the present sample screening positive for NES could also result from the use of a relatively young adult sample. NES peaks during late adolescence until the late twenties [55,59], with university students demonstrating a high prevalence, possibly as a result of greater stress levels [63,64,65]. Given that the mean age of the sample included herein was 26 years, it is logical to conclude that possibly, participants’ age affected the prevalence of NES.

A biological basis also appears to exist, increasing the risk for eating pathology in females, compared with males [66,67], with sex steroid hormones being contributors to the sex-differential risk for eating pathology across the life span. On the other hand, the chronotype is associated with higher eating pathologies in men, indicating that when circadian rhythms are accounted for, men might be more affected [68,69]. Nonetheless, no differences were noted in the prevalence of NES between men and women herein. Some researchers suggested that women might be more affected by NES compared with men [47,70], experiencing more symptoms [71], whereas some studies have revealed a greater prevalence among men [52,54,69].

In the present sample, each increment in the depression score heightened the odds of exhibiting more NES symptoms. Similar associations have been reported previously [46,50,63,72,73], estimating that more than half of the patients with NES exhibit major depressive disorder sometime throughout their lifetime [45]. Nevertheless, individuals with higher levels of depression and psychological distress may often resort to emotional eating as a coping strategy to overcome depression [74,75,76]. Those who meet both NES core criteria, including evening hyperphagia and nocturnal ingestions, are at greater risk for experiencing more severe eating pathologies, including food addiction or binge eating [77]. In parallel, a close association has been suggested to link mood and chronotypes [78,79,80]. In particular, the eveningness chronotype is considered a risk factor for the development and remission of depressive symptoms, anxiety and major depressive disorder [78,81,82,83], independent of insomnia [84]. Although the existence of altered, deficient cognitive–emotional processes have been implicated in this association, other factors associated with eveningness, including unhealthy lifestyle habits, a delayed dim light melatonin onset (DLMO) and the propensity of evening types to addiction may also charge this association [17,85].

A positive association with morningness was observed among participants with an NEQ score ≥ 30. According to Natale et al. [86], regression in ED symptomatology is more likely to be associated with the morningness chronotype. Overall, research suggests that the eveningness pattern is mostly associated with increased NES symptomatology [9,87], with patients experiencing NES presenting a circadian delay regarding food intake and overall daily functioning [9]. For instance, in an early study, Goel [5] noted a delayed phase and reduced amplitudes of the behavioral and neuroendocrine circadian clock of patients with NES, including food intake, ghrelin, cortisol and insulin. However, many studies have also demonstrated that NES can similarly be associated with the morning or the intermediate pattern [88]. Approximately 40% of adults belong to either the morning or the evening circadian type, with most of the population falling along the intermediate chronotype [9,18,88], as seen herein. Furthermore, as Romo-Nava et al. [13] promptly noted, variability in eating behavior across the day or night can also be due to changes in the environment, behavior or mood, all “cycling in parallel” and does not necessarily reflect the definite involvement of the circadian system. In verification of this, when NES is apparent, the exacerbated evening energy intake is further increased during weekends [59,89,90], when the daily environment is changed, compared to the weekdays. According to Lemoine [91] and Sağlam [92], it is not only the sleep/wake cycle that contributes to the chronotype, but mental disorders, mental health and seasonality also seem to modulate chronotype preferences. Thus, a variety of factors that were not accounted for in the present study should be evaluated, aside from the chronotype, in order to explain NES. Last but not least, the PPV of the NEQ when applying the ≥30 threshold (72.7%) [36,50] may also explain the results, as it indicates that approximately 2/3 of the participants who screened positive for NES actually experience the syndrome, with the remaining 1/3 being false positive cases.

Although the relationship between NES and morningness might be a true finding in the present sample, it may also be an artifact due to the relatively low internal validity of the SCRAM questionnaire. The tool is rather new, with few studies using it and even fewer research protocols assessing its validity. Previously, researchers [93] noted that prior to its application, further research is required to test the tool in more generalizable samples, replicating the factor structure, while investigating test–retest reliability, discriminant and predictive validity. Furthermore, the tool does not “measure” one variable but assesses three different ones (morningness, good sleep and depressed mood), using three distinct scales, each including reverse questions as well as forward ones. Thus, the low validity observed herein may be due to inherent structural issues, especially given that the validity of the individual scales was greater compared to that of the total score (sum of scales).

As per Plano et al. [78], several ED aspects appear to be mediated by the circadian system, including mood, meal timing, compulsive behavior and sleep quality. In particular, most patients suffering from NES tend to exhibit an evening chronotype, with greater insomnia disturbances, reported poor sleep [94,95], lower sleep efficacy [94] and sometimes even sleep apnea [58]. Due to these confluences, some researchers argue that it is not clear if NES consists of a psychiatric disorder, a sleep disorder, a delayed circadian eating rhythm or simply a metabolic condition with sleep and mental health aspects [95]. As a result, some scientists have questioned whether NES consists of an ED as evidenced by nosographic construct [78,96,97], whereas others suggest that it may not necessarily follow the “medical model paradigm” of disease entities, assuming the existence of a distinct, underlying mechanism that results in the observed symptomatology [98].

Limitations of the present study include its cross-sectional nature, not allowing for assumptions regarding the development of NES and the use of a convenient sample. Using a larger, more representative sample with a greater number of participants aged over 40 years might have reduced the prevalence of NES symptomatology. However, the present study is the only one assessing NES in a Greek population and the first to translate a tool for NES identification into the Greek language. It would also be helpful if diet records were recorded for the participants herein, as they would provide insight into the intake of energy and nutrients between individuals with aggravated NES symptomatology, as well as between the three different chronotypes. However, as this was an internet-based study, the results would not be valid for an accurate dietary analysis. Furthermore, in the present research, “circadian disruption” has been used as an umbrella term to describe disturbances or dysregulations that negatively affect the circadian clock [99], although the need for a more specific and clear terminology and the evaluation of the degree of disruption is apparent [100].

With sleep disturbance and eating behavior being so closely related, assessing each individual’s chronotype becomes important when evaluating feeding behavior [101]. According to Beauchamp [4], depressive symptoms, suboptimal sleep quality and a strong urge to consume food upon night awakenings consist of key elements of the NES psychopathology network and must be set as primary targets for implementing interventions. As a result, research has shown that personalized weight loss programs designed according to the patient’s chronotype can increase the success rate of obesity management [102]. In parallel, treating chronodisruption may unfold an additional pathway to achieving metabolic health, with a variety of interventions being tested at the moment, including bright light therapy, selective serotonin reuptake inhibitors (SSRIs), psychological counseling (cognitive behavioral therapy) and exogenous melatonin intake [16,61,99,103,104].

## 5. Conclusions

The present study revealed high NES symptomatology in the Greek population. Although the sample size does not allow for generalizations, more research is required to understand if this elevated symptomatology is country-specific or reflects the status of the studied population at a single time point. Furthermore, according to the results, depression appears to affect NES symptomatology to a great degree. The present study revealed that in the sample used herein, NES was associated with the morning chronotype; however, further research is required to validate or refute this observation. However, it is evident that more studies are needed to establish the reliability of the SCRAM questionnaire as a tool for assessing day–night patterns. Nonetheless, NES remains an independent clinical entity requiring further investigation with regard to its pathophysiology, as well as its management. As a result, the need for early NES identification is crucial, and a collective effort aiming to reduce NES symptomatology and its detrimental health effects should be made.

## Figures and Tables

**Table 1:** Participants’ general characteristics (N = 533).

		Men (*n* = 177)	Women (*n* = 356)	Total (N = 533)
	Age (years)	26.5 ± 8.6	26.6 ± 9.7	26.6 ± 9.3
Educational level:	Primary/Secondary/Tertiary (%)	1.7/31.6/59.9/6.8	1.7/18/66.9/13.5	1.7/22.5/75.8
Marital status:	Single/in a relationship/married/ divorced/widowed (%)	56.5/29.4/13/ 1.1/0	37.4/40.7/18.5/ 3.1/0.3	43.7/37.0/16.7/ 2.4/0.2
Anthropometry:	BMI (kg/m^2^)	26.1 ± 4.1	23.3 ± 4.7	23.4 (21.1, 26.5)
	Underweight/normoweight/overweight/obese (%)	1.1/44.6/37.3/16.9	9/64.6/19.4/7	6.4/58.0/25.3/10.3

BMI, Body Mass Index.

**Table 2:** Results of the NEQ and SCRAM questionnaires (N = 533).

	Men (*n* = 177)	Women (*n* = 356)	Total Sample (N = 533)
Total NEQ (0–56)	18.4 ± 7.9	17.8 ± 7.2	18.0 ± 7.4
NEQ ≥ 25 (%)	19.8	16.9	17.8
NEQ ≥ 30 (%)	8.5	7.9	8.1
Total SCRAM (18–90)	48.9 ± 7.7	50.2 ± 7.5	49.7 ± 7.9
SCRAM Good Sleep (6–30)	19.4 ± 4.5	19.3 ± 4.9	19.3 ± 4.8
SCRAM Morningness (6–30)	17.5 ± 5.1	19.4 ± 4.5	17.4 ± 5.1
SCRAM Depression (6–30)	11.9 ± 4.3	13.7 ± 5.0 ***	13.1 ± 4.8

NEQ, Night Eating Questionnaire [36]; SCRAM, Sleep, Circadian Rhythms, and Mood Questionnaire [37]; *** significantly different compared to men, *p* < 0.001.

**Table 3:** NEQ and SCRAM scores and prevalence of NES, by BMI strata (N = 533).

	Underweight (*n* = 34)	Normoweight (*n* = 309)	Overweight (*n* = 135)	Obesity (*n* = 55)	*p*
Total NEQ	18.5 ± 7.2	17.6 ± 7.2	18.6 ± 7.5	18.8 ± 8.4	0.449
Total SCRAM	50.6 ± 8.1	50.0 ± 7.8	49.2 ± 7.9	49.0 ± 8.4	0.631
NEQ ≥ 25 (%)	11.8	15.5	23.0	21.8	0.172
NEQ ≥ 30 (%)	11.8	6.1	9.6	12.7	0.239

BMI, body mass index; NEQ, Night Eating Questionnaire [36]; NES, night eating syndrome; SCRAM, Sleep, Circadian Rhythms, and Mood Questionnaire [37].

**Table 4:** Associations between NES and the SCRAM questionnaire (total and subscales) using logistic regression models.

	Model	OR	95% CI	*p*
NEQ score ≥ 25 vs. total SCRAM	Crude	0.97	0.95–1.00	0.056
	Adjusted ^1^	0.97	0.95–1.00	0.060
	Adjusted ^2^	0.97	0.95–1.00	0.070
NEQ score ≥ 25 vs. Good Sleep	Crude	**0.95**	**0.91–0.99**	**0.047**
	Adjusted ^1^	**0.95**	**0.91–0.99**	**0.047**
	Adjusted ^2^	0.96	0.91–1.00	0.073
NEQ score ≥ 25 vs. Morningness	Crude	1.03	0.98–1.07	0.210
	Adjusted ^1^	1.03	0.98–1.08	0.243
	Adjusted ^2^	1.03	0.98–1.07	0.287
NEQ score ≥ 25 vs. Depression	Crude	**1.05**	**1.01–1.10**	**0.021**
	Adjusted ^1^	**1.06**	**1.01–1.10**	**0.013**
	Adjusted ^2^	**1.06**	**1.01–1.11**	**0.020**
NEQ score ≥ 30 vs. total SCRAM	Crude	0.99	0.96–1.04	0.960
	Adjusted ^1^	0.99	0.96–1.04	0.973
	Adjusted ^2^	1.01	0.96–1.04	0.966
NEQ score ≥ 30 vs. Good Sleep	Crude	0.95	0.89–1.02	0.198
	Adjusted ^1^	0.95	0.89–1.02	0.133
	Adjusted ^2^	0.96	0.90–1.02	0.189
NEQ score ≥ 30 vs. Morningness	Crude	**1.08**	**1.02–1.15**	**0.012**
	Adjusted ^1^	**1.09**	**1.02–1.16**	**0.011**
	Adjusted ^2^	**1.09**	**1.02–1.16**	**0.013**
NEQ score ≥ 30 vs. Depression	Crude	0.99	0.93–1.06	0.922
	Adjusted ^1^	0.99	0.93–1.07	0.957
	Adjusted ^2^	0.99	0.93–1.06	0.844

BMI, body mass index; CI, confidence intervals; NEQ, Night Eating Questionnaire [36]; NES, night eating syndrome; OR, odds ratio; SCRAM, Sleep, Circadian Rhythms, and Mood Questionnaire [37]; ^1^ adjusted for sex, age and tertiary education; ^2^ adjusted for sex, age, tertiary education and BMI.

## Data Availability

The data presented in this study are available on request from the first author. The data are not publicly available due to ethical issues.
